# Environmental influences on familial discordance of phenotype in people with homocystinuria: a case report

**DOI:** 10.1186/1752-1947-2-113

**Published:** 2008-04-20

**Authors:** Francois Maillot, Jan P Kraus, Philip J Lee

**Affiliations:** 1 Charles Dent Metabolic Unit, The National Hospital for Neurology and Neurosurgery, Queen Square, London, UK; 2 CHRU de Tours, Service de Médecine Interne et Nutrition, Tours, France; 3 DNA Diagnostics Laboratory, University of Colorado at Denver and Health Sciences Center, Aurora, USA

## Abstract

**Introduction:**

Non-heritable factors may have an influence on the clinical expression of monogenic inherited metabolic diseases.

**Case presentation:**

This is a case report of a man whose mother had been diagnosed late in childhood with pyridoxine responsive homocystinuria with lens dislocation and neurodevelopmental delay. These severe complications were not observed in her son who was pyridoxine unresponsive but who had been treated appropriately since early infancy.

**Conclusion:**

The phenotype of people with homocystinuria can be discordant within a family, with variability in metabolic and clinical expression depending upon both the genotype and therapeutic interventions. Offspring of people with homocystinuria should be screened in early infancy and, if positive, treated appropriately whether they have pyridoxine responsive or unresponsive disease.

## Background

Homocystinuria is an inherited recessive disorder due to cystathionine beta-synthase (CBS) deficiency, with a wide spectrum of clinical manifestations involving ocular lens dislocation, skeletal disproportion, osteoporosis, vascular thromboses and central nervous system dysfunction. We report a familial discordance of phenotype between a mother and her son, both affected with homocystinuria, to illustrate powerful environmental influences on the metabolic phenotype.

## Case presentation

Our patient, born in 1986, was the second son of a woman who had had progressive loss of vision during the first 7 years of her life, related to bilateral dislocated lenses. She was diagnosed with homocystinuria when both lenses were surgically removed when she was 7 years old. Plasma free homocystine and methionine were 48 μmol/l (*N *= 0) and 670 μmol/l (*N *= 19–25), respectively. She had moderate learning difficulties, a depressed sternum, pes cavus, high arched palate, fair skin with easily seen veins and signs of osteoporosis on spinal X-rays. She had neither arachnodactyly nor thromboembolic events. She was initially and successfully treated with pyridoxine 500 mg/day, which was reduced to 100 mg/day and then 50 mg/day.

At age 24 years, she had a normal pregnancy giving birth to an unaffected child, without post-partum complications. At age 29 years, she had a male child by a different unrelated partner. The plasma total homocysteine (tHcy) in this child was elevated at 107 μmol/l (*N *= 5–15). Follow-up of the mother between the ages of 29 and 48 years showed satisfactory metabolic control (plasma tHcy 21 – 112 μmol/l, plasma methionine 45–48 μmol/l) with therapy including pyridoxine 50 mg/day, folic acid 5 mg/day, cyanocobalamin 50 μg/day and an unrestricted diet. No new clinical events have occurred, despite the fact she was a mild smoker. Molecular study of the CBS gene showed the mutations c.374 G>A (R125Q) in exon 3 and c.671 G>T (R224L) in exon 6.

The second child of our patient was seen at the age of 17 days for homocystinuria screening. His father had no family history of homocystinuria. At the age of 17 days, free Hcy was not detected but 8 months later plasma tHcy and methionine were 107 and 1999 μmol/l, respectively. A methionine-restricted diet was started, with pyridoxine 50 mg/day and folic acid 5 mg/day. Between the age of one and nine years, metabolic control was good with plasma tHcy less than 5–13 μmol/l, methionine 46–53 μmol/l, growth was normal and no lens dislocation was observed. The patient had no arachnodactyly, no bone deformities and his neurological development was normal. At age 10 years, betaine 3 g/day was started. From age 13 to 19 years, plasma tHcy and methionine mostly remained high, at 100–201 and 636–708 μmol/l respectively, despite increasing doses of pyridoxine to 300 mg/day, betaine to 4.5 g/day and adding cyanocobalamin 50 μg/day. No clinical events were reported and eye examination remained normal despite suspected non-compliance with therapy. At age 19 years, his DEXA lumbar spine *Z*-score was -2.7. Molecular analysis of the CBS gene showed the mutations c374 G>A (R125Q) and c.689 insC, which predicts a frameshift and downstream stop codon.

## Discussion and conclusion

For the mother, a low dose of pyridoxine was able to maintain a satisfactory plasma tHcy without dietary methionine restriction. This indicates that she had pyridoxine responsive homocystinuria. Patients homozygous for the R125Q mutation have been previously described as pyridoxine non-responsive [1] or partially pyridoxine responsive if associated with the severe phenotype [2]. Thus, the case of the patient's mother suggests that R125Q mutation in one copy, in association with another missense mutation (R224L), does not result in pyridoxine non-responsiveness. Moreover, we think that her severe phenotype was more related to late diagnosis than her genotype. Her son did not inherit her pyridoxine responsiveness as a high dose of pyridoxine was unable to significantly reduce plasma tHcy [3]. He carried a novel, severe and pathogenic mutation (c.689 insC) of the CBS gene, in association with the maternally inherited R125Q, which resulted in a pyridoxine non-responsive homocystinuria. It is also likely that, after a period of good metabolic control during childhood, he failed to maintain a methionine-restricted diet in the teenage years, a potential problem encountered in adolescence during the follow-up of many patients with inborn errors of metabolism [4]. In most patients, pyridoxine responsiveness is based on the presence of residual activity of mutant CBS, which have been closely linked to CBS mutations [5]. For example, the frequent G370S mutation, in one or two copies, appears to be incompatible with pyridoxine responsiveness, producing a severe phenotype [1]. Conversely, the I278T mutation usually confers pyridoxine responsiveness, whether in homozygotes or compound heterozygotes [6]. In the case presented here, the son has inherited a severe pyridoxine non-responsive mutation from his father who presumably was a heterozygous carrier of this mutant CBS allele. It has been shown that an identical CBS genotype does not always result in the same phenotype, even within a family, and although pyridoxine responsiveness is absolutely constant within sibships, the clinical phenotype is often not [5]. This indicates that non-heritable factors may influence the clinical expression of homocystinuria in genetically predisposed individuals.

Beyond the genotype-phenotype correlation, the homocystinuria described in this family shows that a maximal benefit from therapy may be expected when the disorder is detected in early infancy. Although it is generally recognized that pyridoxine responsive patients are more mildly affected than non-responsive patients, the cases reported here show that a methionine restriction diet started early can prevent or delay a number of important complications of CBS deficiency, in particular neurodevelopment and lens dislocation. This is consistent with previous data showing that patients carrying the G307S mutation seem to have moderate to severe phenotypes, except for those treated from birth [5]. Newborn screening and early treatment for phenylketonuria dramatically alters the clinical phenotype and our cases mirror this. We recommend that offspring of people with homocystinuria should be screened early in infancy and if positive, treated appropriately whether they have pyridoxine responsive or unresponsive disease.

## Competing interests

The authors declare that they have no competing interests.

## Authors' contributions

All of the authors have contributed to the writing of this manuscript, have been involved in subsequent revisions and have given final approval of the version to be published.

## Consent

Written informed consent was obtained from the patients for publication of this case report. A copy of the written consent is available for review by the Editor-in-Chief of this journal.
